# Gain-of-function of the cytokinin response activator ARR1 increases heat shock tolerance in *Arabidopsis thaliana*

**DOI:** 10.1080/15592324.2022.2073108

**Published:** 2022-05-10

**Authors:** Sumudu Karunadasa, Jasmina Kurepa, Jan A Smalle

**Affiliations:** a Department of Plant Biology, Carnegie Institution for Science, Stanford, California, USA; b Department of Plant and Soil Sciences, College of Agriculture Food and Environment, University of Kentucky, Lexington, Kentucky 40546, USA and Kentucky Tobacco Research & Development Center, University of Kentucky, Lexington, Kentucky, USA

**Keywords:** Arabidopsis, abiotic stress, cytokinin, heat shock, heat shock proteins

## Abstract

In addition to its well-established role in plant development, the hormone cytokinin regulates plant responses to biotic and abiotic stresses. It was previously shown that cytokinin signaling acts negatively upon drought and osmotic stress tolerance and that gain-of-function of the cytokinin response regulator ARR1 causes osmotic stress hypersensitivity. Here we show that increased ARR1 action increases tolerance to heat shock and that this is correlated with increased accumulation of the heat shock proteins Hsp17.6 and Hsp70. These results show that the heat shock tolerance of plants can be elevated by increasing the expression of a cytokinin response activator.

## Introduction

Cytokinins (CKs) have been predominantly studied as grown hormones and their roles in numerous aspects of plant development (e.g., formation and function of meristems, the control of leaf and root development, source/sink behavior of cells and organs, and the timing of leaf senescence) has been thoroughly investigated.^1–4^ Recently, with CK biosynthesis and signaling pathways and CK-dependent growth control mechanisms defined,^5–8^ more studies focused on understanding the role of CKs in stress responses. Although these studies implicated CKs in regulating various biotic and abiotic stress responses,^2–4,9–14^ a complete understanding of the role of CKs in the regulation of growth under stress conditions is still lacking. The emerging theme is that the role of CK in abiotic stress tolerance depends on the type of stress plants are exposed to. For example, CKs were shown to reduce drought and osmotic stress tolerance^15–18^ but promote tolerance to temperature stress.^10,19^

An increase in temperature leads to a heat shock response, a fast inducible response that includes the heat shock factors (Hsps)-mediated transcription of genes encoding for cytoprotective heat shock proteins (Hsps).^20,21^ Hsps are molecular chaperones that refold misfolded proteins and are involved in any cellular process that requires the maintenance of proteostasis.^22,23^ Considering that CK is a growth hormone that leads to major changes in protein synthesis,^18,24–27^ it can be expected that at least some of the *Hsp* genes are CK-regulated to aid in the folding of the de novo synthesized CK-induced proteins. It follows that if CK content or action increases, the amount of CK-induced Hsps increases, which may affect the heat shock tolerance. Indeed, CK treatments, mutant analyses, transcriptomics, proteomics, and data mining approaches have shown that CKs are directly involved in the regulation of heat stress tolerance.^28–30^ However, one basic and applied aspect of the CK/heat shock tolerance link has still not been investigated: it has not been shown that plants with a transgenically increased CK action have increased heat shock tolerance. Here, we analyze the heat shock tolerance of Arabidopsis transgenic lines expressing ARR1, an Arabidopsis type-B cytokinin response regulator (RR) that promotes CK responses.^31,32^ The type-B ARRs are phosphorylated during CK signal transduction, and that converts them from a latent into an active form.^6,31–33^ We show that overexpression of the wild-type and activated (phosphomimic) variant of ARR1 increases heat shock tolerance at least partly by the upregulation of Hsps, suggesting that stress tolerance can be engineered by transgenically enhancing CK signaling.

## Results and discussion

**Figure 1.**
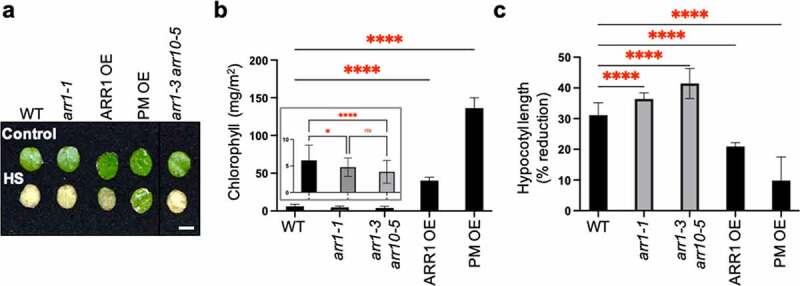
Heat-shock response of ARR1 gain- and loss-of-function lines. (a) Leaf discs excised from mature leaves of 30-day-old plants were floated in MES-KOH buffer (pH 6.8) for 2.5 h at 22°C (control) or 45°C (HS, heat shock) and then incubated in the same buffer at 22°C for 2 days before photography. Representative leaf discs of the Col-0 wild type (WT), *arr1-1* single and double mutant, and two ARR1 gain-of-function lines are shown. Scale bar: 5 mm. (b) Chlorophyll levels in heat-shocked leaf discs. The chlorophyll content is presented as mean ± SD (n ≥ 3). *, P < .05; ****, P < .0001; ns, not significant (one-way ANOVA with Tukey’s multiple comparisons test). The insert shows the chlorophyll levels in the wild-type leaf discs compared to leaf discs of the *arr1-1* and *arr1-3 arr10-5* mutant. (c) Basal seed thermotolerance tests. Sterilized and stratified seeds were exposed to light for 4 h at 22°C (control) or at 45°C (heat shock) and then germinated and grown in darkness at 22°C. Etiolated seedlings were photographed after 3 days of growth, and hypocotyl lengths were measured using ImageJ. The data are presented as the percentage inhibition of hypocotyl length in response to heat shock (mean ± SD; n ≥ 20). **, P < .01; ****, P < .0001 (one-way ANOVA with Tukey’s multiple comparisons test).

It was previously shown that optimal CK sensitivity is required to maintain heat stress tolerance,^28^ indicating a positive role for CK. To test whether increased CK action indeed increases heat shock tolerance, we analyzed the heat-shock responses of transgenic lines overexpressing the CK response activator ARR1 (ARR1 OE)^18^ or the constitutively active phosphomimic ARR1 version (PM OE),^34^ both of which were generated in the *arr1-1* mutant background. In a first response assay, we tested the effect of heat shock on the viability of rosette leaf discs by measuring chlorophyll loss as a marker of the adverse effect of heat shock on chloroplast integrity.^35^ Both ARR1 gain-of-function (GOF) lines (i.e., ARR1 OE and PM OE) were more tolerant to the heat shock treatment and retained more chlorophyll compared to the wild type (Figure 1a, b). There was, however, a difference between the GOF lines; the PM OE line retained more chlorophyll than the ARR1 OE line, suggesting a higher heat shock tolerance level (Figure 1a,b). The CK-resistant mutants *arr1-1* and *arr1-3 arr10-5* (loss-of-function lines, LOF) also responded significantly differently from the wild type and showed a hypersensitive response as evidenced by the accelerated decrease in chlorophyll content (Figure 1b, inset). In a second response assay, we determined the extent to which heat shock pretreatment of seeds leads to inhibition of hypocotyl elongation.^36^ Again, both GOF lines were more tolerant to the heat shock treatment, with the PM OE line displaying a higher tolerance level than ARR1 OE, whereas LOF lines were hypersensitive to heat shock (Figure 1c). Therefore, the results of two physiologically and developmentally different heat shock assays show that the GOF transgenic lines have increased heat shock tolerance.

**Figure 2.**
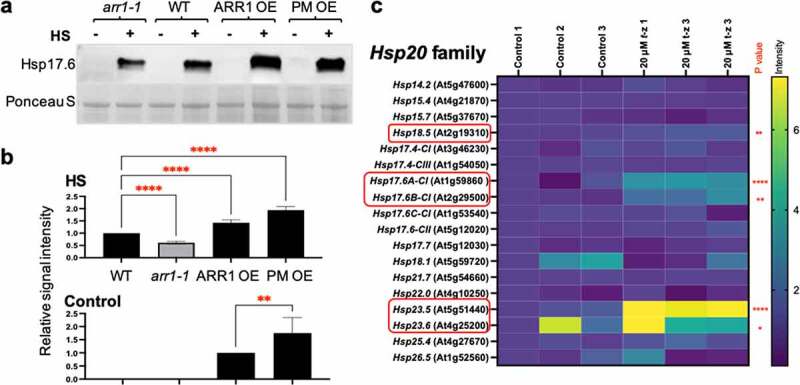
Cytokinin affects Hsp17.6 levels in ARR1 gain- and loss-of-function lines. (a) Representative immunoblot probed with anti-Hsp17.6 antibodies. Seven-day-old seedlings were kept for 2 h at 22°C or at 40°C. Ponceau S-stained membrane region encompassing the large subunit of RuBisCO (LSU) is shown as the loading control. (b) Quantification of the Hsp17.6 levels. Signal intensities of the control samples and the heat-shocked samples were quantified separately as they required quantification at images obtained after different exposure times. Data was normalized to the wild type and shown as mean± SD; n = 3 (**, P < .01; ****, P < .0001; one-way ANOVA with Tukey’s multiple comparisons test). (c) Relative expression levels of the *Hsp20* gene family after 3-hour-long treatment with cytokinin *trans*-zeatin (*t*-z). Microarray data were extracted from publicly available series GSE5698 (AtGenExpress: Cytokinin treatment of seedlings,^39^). Values are presented relative to the control. The significance of expression change between wild type and treatment and the intensity scale are shown on the right. Non-significant differences are not marked and significant changes are marked with an asterisks (*, P < .05; ** < 0.01; ****, P < .0001). Cytokinin-upregulated genes are circled in red.

The heat shock response in plants, as in non-plant species, involves the increased expression of Hsps.^20,21,23^ A previous study described a set of *Hsp* genes that are both heat shock- and CK-regulated.^28^ Most of these genes belong to the *Hsp20* family^28^ and encode small Hsps (sHsps) whose main functions are to unfold proteins, suppress aggregation, and promote protein refolding to a functional state in the ATP-independent manner.^37^ Most notably, CK treatment upregulated the expression of members of the *Hsp17.6* subfamily,^28^ and the overexpression of one of these CK-inducible *Hsp17.6* (*Hsp17.6B-CII*, At2g29500) promotes heat stress tolerance in transgenic plants.^38^ That led us to test the Hsp17.6 levels in control and heat-shocked GOF and LOF lines (Figure 2a,b). The Hsp17.6 proteins were heat shock-induced in all lines, but to a different level: whereas the induction level in *arr1-1* was lower than in the wild type, the induction levels were higher in both GOF lines (Figure 2a,b). In addition, whereas the Hsp17.6 proteins were undetectable in the untreated wild-type and *arr1-1* plants, they were detected in the untreated GOF lines (Figure 2b). Considering that *Hsp17.6* overexpression promotes heat shock tolerance in Arabidopsis,^38^ increase in Hsp17.6 levels in both the untreated and heat shock-treated GOF lines is in agreement with their increased heat shock tolerance. Moreover, the PM OE line had a significantly higher level of Hsp17.6 in untreated seedlings than ARR1 OE (Figure 2a,b), which is in agreement with the higher heat shock tolerance of PM OE (Figure 1). A recent study has shown that the CK transcriptomics response and the transcriptomics response to the expression of a constitutively active form of ARR1 are strikingly similar,^40^ implying that the effects of increased ARR1 action closely resembles the effects of CK treatment. Thus, the CK-dependent regulation of *Hsp* genes is relevant to any phenotypical changes associated with the GOF lines. To test whether *Hsp20* gene family members other than *Hsp17.6* are involved in the CK response, we determined the transcript levels of all *Hsp20* gene family members in plants treated with the CK *trans*-zeatin^39^ (Figure 2c). We selected this gene expression data set for analysis because it involves treatment with a high CK concentration, which is expected to reveal a larger set of CK-regulated genes.^39^ Indeed, it was recently shown that using a higher CK dose for treatments leads to the identification of a larger number of CK- genes.^40^ In addition to *Hsp17.6* genes, *Hsp18.5, Hsp23.5*, and *Hsp23.6* genes were significantly upregulated by CK treatment (Figure 2c).

**Figure 3.**
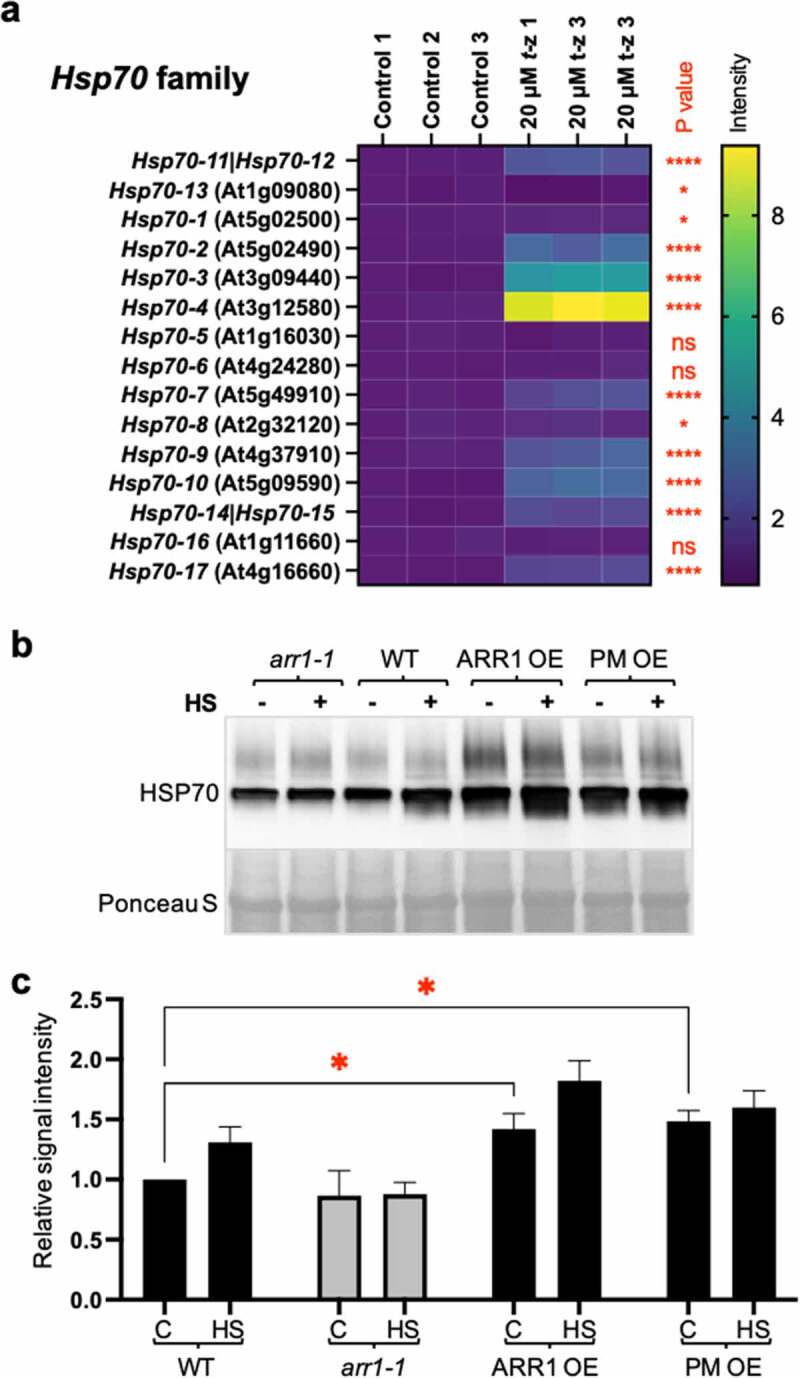
Cytokinin affects Hsp70 levels. (a) Expression levels of the *Hsp70* gene family after 3-hour-long treatment of 21-day-old plants with cytokinin *trans*-zeatin (*t*-z). Values are presented relative to the control. Non-significant differences (ns) are marked. *, P < .05; ****, P < .0001. The AGI code of *Hsp70-11* (BIP1) is At5g28540, *Hsp70-12* (BIP2) is At5g42020, *Hsp70-14* is At1g79930, and *Hsp70-15* is At1g79920. (b) Representative immunoblot probed with anti-Hsp70 antibodies. The experimental conditions as the same as in Figure 2. Ponceau S-stained membrane region encompassing the large subunit of RuBisCO (LSU) is shown as the loading control. (c) Quantification of the Hsp70 levels. Data were normalized to the wild type and shown as mean± SD; n = 3 (* < 0.05; two-way ANOVA with Šídák’s multiple comparisons test).

To test if ARR1 GOF also impacts heat shock tolerance by increasing the expression of other Hsps, we considered Hsp70, a heat shock protein encoded in Arabidopsis by a gene family of 17 members.^41^ Hsp70s are proven to be tightly linked to heat shock tolerance as their overexpression increases heat stress tolerance in transgenic plants,^42–44^ and the combined loss of function of a subset of *Hsp70* genes causes heat stress hypersensitivity.^45^ Because some,^46,47^ but not all^28,31,48–50^ transcriptomic studies of CK-inducible gene sets listed an *Hsp70* family member as a CK-regulated gene, we re-analyzed the effect of CK on the transcription of all *Hsp70* genes. This analysis revealed that most *Hsp70* genes are significantly upregulated by a three-hour-long CK treatment, while one gene (*Hsp70-13*) is CK repressed, and three *Hsp* genes are not affected (Figure 3a). This suggested that the upregulation of Hsp70 protein levels is an important part of the CK response.

To test whether we can detect changes in Hsp70s levels in GOF and LOF lines, we used anti-Hsp70 antisera raised against a synthetic peptide conserved in all higher plant Hsp70 proteins, which allowed us to recognize the total change in Hsp70 levels. Hsp70 levels were increased in both untreated GOFs, whereas the decrease in *arr1-1* was trending but not significantly lower compared to the wild type (Figure 3b, c). Hsp70s were induced by heat shock in the wild type, but no induction was apparent in the *arr1-1* mutant, suggesting that heat shock induction of Hsp70s requires a functional CK response pathway (Figure 3b,c). The elevated Hsp70 protein level in GOF lines was not further increased by a two-hour heat shock, indicating that the increased CK action raised the Hsp70 level to a maximum (Figure 3b,c). Contrary to the different upregulation levels of Hsp17.6 in the GOF lines, the Hsp70 increase was nearly identical, suggesting that the increased CK action in both of these lines was sufficient to raise the Hsp70 level to this maximum. We concluded that the heat shock tolerance conferred by increased ARR1 action involves the increased expression of genes belonging to at least two *Hsp* gene families, namely *Hsp20* and *Hsp70*.

## Summary

We have shown that the gain of function of the CK response activator ARR1 enhances heat shock tolerance and that this is – at least in part – mediated by the increased accumulation of Hsp17.6 and Hsp70. Since both the CK signaling and heat shock tolerance mechanisms are conserved throughout higher plants,^5,20^ we suggest that increasing CK action is a promising approach for improving the heat shock tolerance of agriculturally important plant species.

## Materials and methods

### Plant materials and growth conditions

The lines used were Arabidopsis (*Arabidopsis thaliana* L. Heynh) Columbia 0 (Col-0) as the wild-type, and the Col-0 mutants *arr1-1*^51^ (a gift from Atsuhiro Oka) and *arr1-3 arr10-5*^31^ (obtained from the ABRC Seed Stock Center). The wild-type ARR1 overexpression line (ARR1 OE) and phosphomimic ARR1 expression line (PM OE) in the *arr1-1* mutant background were previously described.^34,52^ For all experiments, seeds were surface sterilized, stratified for one day, and plated on half-strength Murashige and Skoog medium containing 1% sucrose and 0.8% PhytoAgar (MS/2, pH 5.7). Plants were grown in a growth chamber at 22°C under continuous light (80 mmol m^−2^ s^−1^).

## Heat shock treatments and immunoblotting

Seedlings were grown for seven days on MS/2 media in the growth chamber set at 22°C. Half of the samples were then transferred to an illuminated incubator set at 40°C and heat-shocked for two hours. For protein extraction, seedlings were weighed, three volumes of 2x Laemmli sample buffer were added, and tissue was disrupted using zirconium beads in a bead beater. After pelleting the debris, proteins were separated on 4–20% SDS-PAGE gels (Bio-Rad) and transferred to nitrocellulose membranes. Membranes were blocked using 10% fat-free milk and washed with PBS containing 0.2% Tween‐20. Antibodies used were anti-Hsp17.6 (1:5,000 dilution; Agrisera Product no AS07 254), anti-Hsp70 (1:5,000; Agrisera Product no AS08 371), and horseradish peroxidase-conjugated goat anti-rabbit antisera (1:1000; Santa Cruz, CA, USA). Immunoblots were developed using SuperSignal West Femto substrate (Thermo-Pierce, Rockford, IL, USA) using a ChemiDoc XRS molecular imager and quantified using Quantity One® software (Bio-Rad).

## Heat shock response assays

Basal seed thermotolerance was tested using the protocol described by Hong and Vierling.^36^ In brief, the seeds were sterilized, sown on MS/2 media, stratified, and either exposed to light for 4 hours at 45°C (heat-shocked samples) or exposed to light at 22°C (control samples). The plates were then wrapped in aluminum foil and transferred to a growth chamber set at 22°C, where they were kept for three days. The etiolated seedlings were photographed, and their hypocotyl lengths were measured by using ImageJ software.

In the leaf disc assay, the leaf discs were punched out from mature leaves of 30-day-old soil-grown plants transferred to 12-well cell culture plates with 10 mM MES-KOH buffer, pH 6.8 (2 ml per well). Control samples were kept at 22°C, and the test samples were kept at 45°C for 2.5 hours. Heat-shocked samples were then returned to 22°C, and leaf discs were photographed after two days. The chlorophyll content was measured using a CCM 300 chlorophyll content meter (Opti-Sciences).

## Statistical and transcriptomic analyses

Descriptive statistics, plotting, and hypothesis testing were done by using Prism 9.3 (GraphPad Software Inc., La Jolla, CA, USA). All data are presented as mean ± SD. The number of replicates and statistical tests used for the analyses are indicated in Figure Legends. The publicly available series GSE5698 (AtGenExpress: Cytokinin treatment of seedlings^39^) was obtained from the Gene Expression Omnibus (GEO) database at the National Center for Biotechnology Information (NCBI). GEO2R was used to obtain sample values used for heat map generation. The sample values were normalized to wild-type control, analyzed, and displayed using Prism 9.3.
